# Mental health service preferences of patients and providers: a scoping review of conjoint analysis and discrete choice experiments from global public health literature over the last 20 years (1999–2019)

**DOI:** 10.1186/s12913-021-06499-w

**Published:** 2021-06-18

**Authors:** Anna Larsen, Albert Tele, Manasi Kumar

**Affiliations:** 1 Department of Global Health, University of Washington, Seattle, WA 98195 USA; 2 IKUZE AFRICA, Nairobi, 00100 Kenya; 3 Department of Psychiatry, University of Nairobi, (47074), Nairobi, 00100 Kenya

**Keywords:** Conjoint analysis, Discrete choice experiments, Mental health interventions

## Abstract

**Background:**

In designing, adapting, and integrating mental health interventions, it is pertinent to understand patients’ needs and their own perceptions and values in receiving care. Conjoint analysis (CA) and discrete choice experiments (DCEs) are survey-based preference-elicitation approaches that, when applied to healthcare settings, offer opportunities to quantify and rank the healthcare-related choices of patients, providers, and other stakeholders. However, a knowledge gap exists in characterizing the extent to which DCEs/CA have been used in designing mental health services for patients and providers.

**Methods:**

We performed a scoping review from the past 20 years (2009–2019) to identify and describe applications of conjoint analysis and discrete choice experiments. We searched the following electronic databases: Pubmed, CINAHL, PsychInfo, Embase, Cochrane, and Web of Science to identify stakehold,er preferences for mental health services using Mesh terms. Studies were categorized according to pertaining to patients, providers and parents or caregivers.

**Results:**

Among the 30 studies we reviewed, most were published after 2010 (24/30, 80%), the majority were conducted in the United States (11/30, 37%) or Canada (10/30, 33%), and all were conducted in high-income settings. Studies more frequently elicited preferences from patients or potential patients (21/30, 70%) as opposed to providers. About half of the studies used CA while the others utilized DCEs. Nearly half of the studies sought preferences for mental health services in general (14/30, 47%) while a quarter specifically evaluated preferences for unipolar depression services (8/30, 27%). Most of the studies sought stakeholder preferences for attributes of mental health care and treatment services (17/30, 57%).

**Conclusions:**

Overall, preference elicitation approaches have been increasingly applied to mental health services globally in the past 20 years. To date, these methods have been exclusively applied to populations within the field of mental health in high-income countries. Prioritizing patients’ needs and preferences is a vital component of patient-centered care – one of the six domains of health care quality. Identifying patient preferences for mental health services may improve quality of care and, ultimately, increase acceptability and uptake of services among patients. Rigorous preference-elicitation approaches should be considered, especially in settings where mental health resources are scarce, to illuminate resource allocation toward preferred service characteristics especially within low-income settings.

**Supplementary Information:**

The online version contains supplementary material available at 10.1186/s12913-021-06499-w.

## Background

Mental disorders are the leading cause of disability and the second leading cause of death globally, accounting for over 276 million disability-adjusted life years and leading to over 9 million deaths annually [1]. The burden of depression, anxiety, substance use, and some neurological disorders is comparable to noncommunicable diseases like cancer and coronary heart disease, more prominently known for their worldwide health impact [2]. Despite this burden, mental health services are scarce in many areas of the world, especially low-and-middle-income countries [3]. Even when services exist, they may not serve patient and provider needs and be based on either of their preferences to optimize formal health care services.

There is strong evidence from other disease areas (e.g., cancer, HIV, and veteran health services, among others) that services which engage patients from the beginning – during conceptualization of the service – can be highly successful and effective [4]. The global impetus from the Sustainable Development Goals (SDGs) Universal Health Coverage initiative (SDG 3) focuses on the need for services that are accessible, affordable, good quality and acceptable by people for whom these are designed [5]. Correspondingly, taking example of services for adolescents and youth, the World Health Organization (WHO) encourages service provision that is responsive to patient preferences, such as “youth-friendly services” described in the Global Accelerated Action for Health of Adolescents (AA-HA!) guidelines, to encourage uptake and engagement in services [6]. The WHO considers patient-centeredness not only integral to human rights enforcement in health services but also central to developing integrated systems [7].

As mental ill-health becomes increasingly recognized as a global burden, innovations are emerging to provide accessible, affordable, and acceptable prevention, care, and treatment services to the diverse populations faced with mental health issues [8–10]. Information and messages about mental health, preventative services, treatment characteristics, provider approaches, and care provision modalities must continue to evolve based on stakeholder preferences to ensure relevance and desirability. However, patient involvement in shaping mental health practice has been minimal, especially in low-resource settings [11–14].

Despite establishing the need to rigorously elicit patient preferences for healthcare, *“precisely how to systematically assess and incorporate patient preferences in the clinical setting remains an area with a need for methodological development”* (astutely articulated by Wittink et al) [15]. Multiple methods have been developed and applied to empirically identify preferences. Two widely used quasi-experimental, quantitative approaches made popular by their use in market research and grounded in macroeconomic principles [16] are conjoint analysis (CA) and discrete choice experiments (DCE) DCEs [17–19]. Both methods offer rigorous and systematic approaches for eliciting preferences for service or product attributes from customers and stakeholders [20].

Conjoint analyses decompose an intervention into its key attributes, then pose the attributes to patients to understand patient-determined values for each attribute [21, 22]. Similarly, in discrete choice experiments, researchers construct treatment or service options from a set of attributes and posing them to patients in an experimental design to enable independent assessment of preferences for specific attributes in statistical analysis [23]. The methods are grounded in the premise that goods and services are comprised of discrete attributes and that consumers holistically value goods and services based on the collective levels of the attributes [18]. As such, these methods involve posing options for attributes of services to a stakeholder group who select preferred options from a series of choices that pit attributes against each other. Ultimately, conjoint analysis and discrete choice experiments allow for estimation of the relative importance of aspects of the service, trade-offs between attributes made by stakeholders, and overall service satisfaction based on stakeholder preferences.

These methods are increasingly applied to healthcare settings to enable patient input for patient-centered care [18]. CA and DCEs have been successfully applied for patient preference elicitation in multiple areas of healthcare, including provider-interactions, health service delivery content and format, and treatment options [18]. Increasingly, CA and DCE methods are applied to mental health service delivery and treatment options. DCEs.

Appropriate and acceptable presentations of mental health services differ between groups such that cultural adaptations should be made for optimal effectiveness [24, 25]. Especially in settings where few mental health services exist, development of novel albeit multimodal services should directly involve patient informed service development. Additionally, understanding preferences may elucidate patient perception of risks and causes of mental disorder, as well as social determinants driving mental health outcomes. In this way, CA and DCEs offer opportunities to further scientific understanding of mental health underpinnings within communities while illuminating gaps in patient knowledge worthy of attention. CA and DCEs offer rigorous and evidence-based approaches to improving acceptability and reducing barriers to mental health services, especially among hard-to-reach populations.

Despite the utility of CA and DCE methods toward improving mental health services, no studies have systematically synthesized information about application of CA and DCE toward preferences in mental health care provision. Understanding where such studies have occurred geographically, the mental health issues to which they were applied, and service and treatment attributes investigated would help identify gaps for further exploration. Further, systematically evaluating the study design components such as the preparatory work utilized, number and type of choices and attributes used, and other methodologic and analytic characteristics may facilitate application of CA and DCE for eliciting preferences in new populations and settings.

Due to the rapid developments in the application of CA and DCEs toward healthcare, specifically for mental health, we considered it timely to conduct a scoping review on applications of CA and DCEs for soliciting and identifying stakeholder preferences for mental health services within the past 20 years globally. We think there is a need to promote their use in global mental health with a focus on LMICs.

DCEs.

Through this scoping review we identified published examples of CA and DCEs for mental health within the literature and mapped their characteristics with the ultimate goal of informing future preference elicitation for mental health services.

## Methods

### Identification of eligible studies and search strategy

We performed a broad search of the literature to identify articles depicting use of CA and DCEs to identify patient and stakeholder preferences for mental health services. Six databases were systematically consulted: Pubmed, CINAHL, PsychInfo, Embase, Cochrane, and Web of Science. Prior to conducting the search, we identified keywords and search terms and organized them appropriately for each database (see Supplementary Table 1). We performed the scoping search in July 2019, yielding 695 total citations (CINAHL: 63, Cochrane: 64, EMBASE: 355, PsychInfo: 61, Pubmed: 67, Web of Science: 85). Endnote X7 Reference Manager was used to manage citations identified. After duplicates (*n* = 160) and citations published before 1990 (*n* = 2) were removed, 533 citations remained. The PRISMA 2020 Statement Preferred Reporting Items for Systematic reviews and Meta-Analyses extension for Scoping Reviews (*PRISMA*-*ScR*) guidelines were followed for this review [26, 27].

### Selection of literature

A two-phased approach was used to identify articles included in the review. In phase 1, all 533 article titles and abstracts were assessed by a single reviewer for their consistency with inclusion/exclusion criteria (see Table 1). Articles were included that utilized CA and DCEs methods and sought preferences for mental health service aspects. We excluded articles that did not utilize these methods or that sought preferences for services not related to mental health, as well as non-English language publications. All articles that did not fit the inclusion criteria were excluded. The main reason for exclusion at the full-text review phase was due to CA and DCEs being non-mental health focused.

During phase 2, the remaining articles were reviewed in full-text separately but in parallel by two reviewers for their consistency with inclusion/exclusion criteria. During this phase, articles without full text versions and student dissertations or theses were additionally excluded. Any remaining reviewer disagreement was resolved with collective review of full-text articles and discussion about relevance. Both reviewers had to agree for an article to be excluded. Overall, 30 articles fit scoping review criteria and were identified for synthesis.

### Data extraction

To address our research objective of investigating the applications of CA and DCEs to ascertain key stakeholder preferences for mental health services, understanding individual level service needs and demand characteristics we systematically examined each article for the population studied, geographical location, sample size, mental health service preferences assessed, methods used to design the study, methods used to analyze preferences, and categories/sub-categories of choices presented. Categories for data extraction were informed by a checklist for developing CA applied to health care settings from the International Society for Pharmacoeconomics and Outcomes Research which helps explain the utility of these methods toward health care improvement (see Table 2). We extracted this information into a comprehensive matrix and assessed the information for emerging patterns and gaps in the utilization of conjoint analysis to evaluate stakeholder preferences for mental health services within existing literature.

**Table 1:** Inclusion/Exclusion criteria for scoping review

Phase	Reviewers	Inclusion Criteria	Exclusion Criteria
1: Titles/abstracts reviewed	Single reviewer	• Conjoint analysis methods used • Preferences for mental health services assessed (e.g., content, format, practitioners, treatment options, etc)	• Non-conjoint analysis methods used • Preferences for non-mental health services assessed • Non-English language
2: Full-text articles reviewed	Two, parallel reviewers	*Same as above*	• *Same as above* • Articles without accompanying full texts • Dissertation or thesis

**Table 2:** Conjoint analysis applications in health: a checklist offered by the International Society for Pharmacoeconomics and Outcomes Research [20]

*Research question and hypothesis clarity:* A sound and testable hypothesis underpinning a research question that is focusing on some aspect of patient health or care. This hypothesis has to be back by existing literature and grounded through some formative qualitative research. *Understanding attributes and levels:* Conjoint analysis focuses on elicitation of preferences or values over the range of attributes and levels that define key domains in the conjoint-analysis tasks. The attribute levels should encompass the range that may be salient to participants, even if those levels are hypothetical or not feasible in each context. *Construction of tasks:* “Tasks” describe the choice options presented to patients from which they make their selected preference. Within a choice task, attributes and levels may be offered individually or in “profiles” where multiple attributes and levels are offered together to represent a service or product option. Thoughtful construction of tasks is helpful to understand trade-offs better. *Experimental nature of the design:* The goal of a conjoint-analysis experimental design is to create a set of tasks that will yield as much statistical information as possible for estimating unbiased, precise preference parameters. In accordance with the experimental nature, the design must be balanced at each level and attribute. *Preference elicitation:* Offering participants contextual information including motivation and explanation for the tasks helps in eliciting the right choices. It is critical that the overall design is not cognitively or semantically challenging for the participants. Pretests and expert, key stakeholder consultation is critical here. Table 2 is taken directly from: Bridges [1]

**Table 3:** Characteristics of studies using conjoint analysis to elicit stakeholder preferences for mental health services

Characteristic	Number (%)	Studies
**Study participants**^a^		
Patients	21 (70%)	Dwight-Johnson et al. (2004), Flach et al. (2004), Townend (2000), Townend et al. (2002), Dwight-Johnson et al. (2013), Wittink et al. (2010), Lee et al. (2014), Fahey et al. (2017), Albus et al. (2005), Ng-Mak et al. (2018), Zimmermann et al. (2013), Bell et al. (2010), Hajime et al. (2018), Huang et al. (2014), Becker et al. (2016), Herman et al. (2016), Cunningham et al. (2017), Okumura et al. (2012), Cunningham et al. (2014), Dwight-Johnson et al. (2010), Zipursky et al. (2017)
Providers	4 (13%)	Riepe et al. (2017), Becker et al. (2016), Cunningham et al. (2018), Cunningham et al. (2012)
Parents/caregivers	7 (23%)	Wymbs et al. (2018), Fegert et al. (2011), Waschbusch et al. (2011), Becker et al. (2016), Cunningham et al. (2015), Cunningham et al. 92,013), Cunningham et al. (2008)
**Country**		
USA	11 (37%)	Wymbs et al. (2018), Dwight-Johnson et al. (2004), Flach et al. (2004), Dwight-Johnson et al. (2013), Wittink et al. (2010), Lee et al. (2014), Waschbusch et al. (2011), Ng-Mak et al. (2018), Huang et al. (2014), Dwight-Johnson et al. (2010), Herman et al. (2016)
Canada	10 (33%)	Bell et al. (2010), Zipursky et al. (2017), Becker et al. (2016), Cunningham et al. (2018), Cunningham et al. (2017), Cunningham et al. (2015), Cunningham et al. (2014), Cunningham et al. (2013), Cunningham et al. (2012), Cunningham et al. (2008)
Germany	4 (13%)	Riepe et al. (2017), Albus et al. (2005), Zimmermann et al. (2013)
UK	3 (10%)	Townend (2000), Townend et al. (2002), Fahey et al. (2017)
Japan	2 (7%)	Hajime et al. (2018), Okumura et al. (2012)
**Year**		
2000–2010	9 (30%)	Townend (2000), Townend et al. (2002), Dwight-Johnson et al. (2004), Flach et al. (2004), Albus et al. (2005), Cunningham et al. (2008), Wittink et al. (2010), Bell et al. (2010), Dwight-Johnson et al. (2010)
2011–2019	21 (70%)	Fegert et al. (2011), Waschbusch et al. (2011), Okumura et al. (2012), Dwight-Johnson et al. (2013), Zimmermann et al. (2013), Cunningham et al. (2013), Lee et al. (2014), Huang et al. (2014, Cunningham et al. (2014), Cunningham et al. (2015), Becker et al. (2016), Herman et al. (2016), Riepe et al. (2017), Fahey et al. (2017), Zipursky et al. (2017), Cunningham et al. (2017), Wymbs (2018), Ng-Mak et al. (2018), Hajime (2018), Cunningham et al. (2018)
**Method**		
Conjoint analysis	16 (53%)	Townend (2000), Townend et al. (2002), Dwight-Johnson et al. (2004), Flach et al. (2004), Albus et al. (2005), Bell et al. (2010), Dwight-Johnson et al. (2010), Okumura et al. (2012), Dwight-Johnson et al. 2013), Zimmermann et al. (2013), Lee et al. (2014), Huang et al. (2014), Riepe et al. (2017), Fahey et al. (2017), Wymbs et al. (2018), Hajime et al. (2018)
DCE	14 (47%)	Cunningham et al. (2008), Wittink et al. (2010), Fegert et al. (2011), Waschbusch et al. (2011), Cunningham (2012), Cunningham et al. (2013), Cunningham et al. (2014), Becker et al. (2016), Herman et al. (2016), Zipursky et al. (2017), Cunningham et al. (2017), Ng-Mak et al. (2018), Cunningham et al. (2018), Flach et al. (2004)
**Mental health issue**		
Mental health general	14 (47%)	Townend (2000), Townend et al. (2002), Albus et al. (2005), Cunningham et al. (2008), Cunningham et al. (2013), Lee et al. (2014), Cunningham et al. (2014), Cunningham et al. (2015), Becker et al. (2016), Herman et al. (2016), Zipursky et al. (2017), Cunningham et al. (2017), Hajime et al. (2018), Cunningham et al. (2018)
Unipolar depression	8 (27%)	Dwight-Johnson et al. (2004), Wittink et al. (2010), Bell et al. (2010), Dwight-Johnson et al. (2010), Okumura et al. (2012), Dwight-Johnson et al. (2013), Zimmermann et al. (2013), Riepe et al. (2017)
ADHD	3 (10%)	Fegert et al. (2011), Waschbusch et al. (2011), Wymbs et al. (2018)
Addiction/substance use	2 (7%)	Flach et al. (2004), Cunningham et al. (2012)
Dementia	2 (7%)	Huang et al. (2014), Fahey et al. (2017)
Bipolar disorder	1 (3%)	Ng-Mak et al. (2018)
**Attributes investigated**		
Adult care and treatment	17 (57%)	Townend (2000), Townend et al. (2002), Dwight-Johnson et al. (2004), Flach et al. (2004), Wittink et al. (2010), Dwight-Johnson et al. (2010), Fegert et al. (2011), Waschbusch et al. (2011), Okumura et al. (2012), Cunningham et al. (2012), Dwight-Johnson et al. (2013), Zimmermann et al. (2013), Lee et al. (2014), Herman et al. (2016), Riepe et al. (2017), Zipursky et al. (2017)
Child mental health interventions	4 (13%)	Cunningham et al. (2008), Cunningham et al. (2013), Cunningham et al. (2015), Cunningham et al. (2018)
Early mental health intervention services/prevention services	2 (7%)	Becker et al. (2016), Hajime et al. (2018)
Messaging/information	2 (7%)	Bell et al. (2010), Cunningham et al. (2014)
Campus/school/community-based services	2 (7%)	Cunningham et al. (2017), Wymbs et al. (2018)
Psychosocial support services	1 (3%)	Albus et al. (2005)
Genetic testing	1 (3%)	Huang et al. (2014)
Pharmacologic attributes	1 (3%)	Ng-Mak et al. (2018)

^a^Percentages may not add to 100% in cases where categories were not mutually exclusive

**Table 4:** Methodologic design employed by conjoint analyses/discrete choice experiments to elicit stakeholder preferences for mental health services

Design Aspect	Specification	N (%)	Studies
Preparatory^a^ work	Literature review	13 (43%)	Dwight-Johnson et al. (2013), Dwight-Johnson et al. (2010), Ng-Mak (2018), Wymbs (2018), Townend (2000), Fach et al. (2004), Fegert et al. (2011), Okumura et al. (2012), Lee et al. (2014), Huang et al. (2014), Becker et al. (2016), Fahey et al. (2017), Zipursky et al. (2017)
	Patient qualitative work	21 (70%)	Albus et al. (2005), Bell et al. (2010), Cunningham et al. (2008), Cunningham et al. (2012), Cunningham et al. (2013), Cunningham et al. (2014), Cunningham et al. (2018), Cunningham et al. (2015), Dwight-Johnson et al. (2013), Dwight-Johnson et al. (2010), Dwight-Johnson et al. (2004), Herman et al. (2016), Ng-Mak et al. (2018), Wittink et al. (2010), Wymbs (2018), Zimmerman et al. (2013), Townend (2000), Townend et al. (2002), Fegert et al. (2011), Okumura et al. (2012), Becker et al. (2016)
	Policy maker/provider qualitative work	3 (10%)	Cunningham et al. (2017), Cunnigham et al. (2018), Becker et al. (2016)
	Quantitative work	15 (50%)	Albus et al. (2005), Cunningham et al. (2008), Cunningham et al. (2012), Cunningham et al. (2013), Cunningham et al. (2014), Cunningham et al. (2017), Cunningham et al. (2018), Dwight-Johnson et al. (2013), Dwight-Johnson et al. (2010), Ng-Mak et al. (2018), Riepe et al. (2017), Wymbs (2018), Zimmerman et al. (2013), Townend et al. (2002), Becker et al. (2016)
	Not specified	2 (7%)	Waschbusch et al. (2011), Hajime (2018)
Type of Choice	Binary	12 (40%)	Ng-Mak et al. (2018), Zimmerman et al. (2013), Townend et al. (2002), Dwight-Johnson et al. (2004), Wittink et al. (2010), Bell et al. (2010), Dwight-Johnson et al. (2010), Fegert et al. (2011), Dwight-Johnson et al. (2013), Lee et al. (2014), Cunningham et al. (2015), Hajime (2018)
	Ternary	16 (53%)	Albus et al. (2005), Becker et al. (2016), Cunningham et al. (2008), Cunningham et al. (2012), Cunningham et al. (2013), Cunningham et al. (2014), Cunningham et al. (2017), Cunningham et al. (2018), Herman et al. (2016), Wymbs (2018), Zipursky et al. (2017), Flach et al. (2004), Wittink et al. (2010), Waschbusch et al. (2011), Okumura et al. (2012), Fahey et al. (2017)
	Not specified	4 (13%)	Townend (2000), Huang et al. (2014), Becker et al. (2016), Riepe et al. (2017)
Number of Attributes	2	1 (3%)	Flach et al. (2004)
	3	2 (7%)	Okumura et al. (2012), Huang et al. (2014)
	4	11 (37%)	Albus et al. (2005), Becker et al. (2016), Cunningham et al. (2008), Cunningham et al. (2012), Cunningham et al. (2013), Cunningham et al. (2014), Cunningham et al. (2017), Cunningham et al. (2018), Wittink et al. (2010), Dwight-Johnson et al. (2013), Fahey et al. (2017)
	5	1 (3%)	Lee et al. (2014)
	6	4 (13%)	Ng-Mak et al. (2018), Dwight-Johnson et al. (2004), Fegert et al. (2011), Hajime (2018)
	7	1 (3%)	Dwight-Johnson et al. (2010),
	8	2 (7%)	Herman et al. (2016), Townend et al. (2002)
	More than 8	12 (40%)	Bell et al. (2010), Waschbusch et al. (2011), Cunningham et al. (2013), Cunningham et al. (2014), Cunningham et al. (2017), Cunningham et al. (2018), Cunningham et al. (2015), Flach et al. (2004), Wittink et al. (2010), Wymbs (2018), Zimmerman et al. (2013), Zipursky et al. (2017),
	Not specified	3 (10%)	Townend (2000), Becker et al. (2016), Riepe et al. (2017)
Number of choices per individual	5 or less	8 (27%)	Cunningham et al. (2008), Cunningham et al. (2013), Fahey et al. (2017), Herman et al. (2016), Okumura et al. (2012), Townend et al. (2002), Zimmerman et al. (2013), Zipursky et al. (2017)
	6–10	7 (23%)	Albus et al. (2005), Dwight-Johnson et al. (2010), Flach et al. (2004), Fegert et al. (2011), Dwight-Johnson et al. (2013), Lee et al. (2014), Fahey et al. (2017)
	11–15	3 (10%)	Dwight-Johnson et al. (2004), Huang et al. (2014), Hajime (2018)
	More than 15	10 (33%)	Becker et al. (2016), Cunningham et al. (2008), Huang et al. (2014), Wittink et al. (2010), Bell et al. (2010), Waschbusch et al. (2011), Cunningham et al. (2012), Cunningham et al. (2017), Cunningham et al. (2015), Cunningham et al. (2018)
	Not specified	3 (10%)	Townend (2000), Riepe et al. (2017), Ng-Mak et al. (2018)
Administration of survey	Self-completed questionnaire	24 (80%)	Albus et al. (2005), Cunningham et al. (2008), Cunningham et al. (2018), Cunningham et al. (2012), Cunningham et al. (2013), Cunningham et al. (2014), Cunningham et al. (2015), Cunningham et al. (2017), Dwight-Johnson et al. (2004), Becker et al. (2016), Herman et al. (2016), Townend (2000), Townend et al. (2002), Waschbusch et al. (2011), Zimmerman et al. (2013), Wittink et al. (2010), Bell et al. (2010), Fegert et al. (2011), Okumura et al. (2012), Huang et al. (2014), Zipursky et al. (2017), Wymbs (2018), Ng-Mak et al. (2018), Hajime (2018)
	Interview administered	5 (17%)	Fahey et al. (2017), Lee et al. (2014), Riepe et al. (2017), Dwight-Johnson et al. (2010), Dwight-Johnson et al. (2013)
Sample size	10–100	8 (27%)	Townend (2000), Townend et al. (2002), Dwight-Johnson et al. (2004), Flach et al. (2004), Wittink et al. (2010), Dwight-Johnson et al. (2013), Riepe et al. (2017), Wymbs (2018)
	101–300	11 (37%)	Albus et al. (2005), Bell et al. (2010), Fegert et al. (2011), Waschbusch et al. (2011), Zimmermann et al. (2013), Lee et al. (2014), Huang et al. (2014), Fahey et al. (2017), Zipursky et al. (2017), Ng-Mak et al. (2018), Hajime (2018)
	> 300	12 (40%)	Cunningham et al. (2008), Dwight-Johnson et al. (2010), Okumura et al. (2012), Cunningham et al. (2012), Cunningham et al. (2013), Cunningham et al. (2014), Cunningham et al. (2015), Becker et al. (2016), Herman et al. (2016), Cunningham et al. (2017), Cunningham et al. (2018), Okumura et al. (2012)
Design Plan	Main effects only	4 (13%)	Townend et al. (2002), Dwight-Johnson et al. (2004), Fegert et al. (2011), Lee et al. (2014)
	Main effects + Interactions	17 (57%)	Albus et al. (2005), Cunningham et al. (2008), Cunningham et al. (2012), Becker et al. (2016), Cunningham et al. (2013), Cunningham et al. (2014), Cunningham et al. (2017), Cunningham et al. (2018), Cunningham et al. (2015), Herman et al. (2016), Ng-Mak et al. (2018), Waschbusch et al. (2011), Wittink et al. (2010), Zimmerman et al. (2013), Zipursky et al. (2017), Dwight-Johnson et al. (2010), Riepe et al. (2017)
	Not clearly reported in the methods but main effects only reported	6 (20%)	Dwight-Johnson et al. (2013), Flach et al. (2004), Bell et al. (2010), Fahey et al. (2017), Wymbs (2018), Hajime (2018)
	Not clearly reported in the methods and unclear in the results	2 (7%)	Huang et al. (2014), Townend (2000)
Analytic software	Sawtooth software	15 (50%)	Albus et al. (2005), Becker et al. (2016), Bell et al. (2010), Cunningham et al. (2008), Cunningham et al. (2012), Cunningham et al. (2013), Cunningham et al. (2014), Cunningham et al. (2015), Cunningham et al. (2017), Cunningham et al. (2018), Herman et al. (2016), Ng-Mak et al. (2018), Waschbusch et al. (2011), Zimmerman et al. (2013), Wymbs (2018)
	Latent gold choice	4 (13%)	Becker et al. (2016), Cunningham et al. (2017), Cunningham et al. (2015), Zipursky et al. (2017)
	SPEED	2 (7%)	Townend et al. (2002), Fegert et al. (2011)
	SPSS	6 (20%)	Dwight-Johnson et al. (2013), Fahey et al. (2017), Lee et al. (2014), Dwight-Johnson et al. (2004), Lee et al. (2014), Hajime (2018)
	SAS	4 (13%)	Dwight-Johnson et al. (2010), Huang et al. (2014), Ng-Mak et al. (2018), Wittink et al. (2010)
	Stata	1 (3%)	Flach et al. (2004)
	Mplus	1 (3%)	Okumura et al. (2012)
	R	1 (3%)	Okumura et al. (2012)
	Atlas.ti	1 (3%)	Ng-Mak et al. (2018)
	MATLAB	1 (3%)	Riepe et al. (2017)
	Not Reported	1 (3%)	Townend (2000)

^a^Percentages may not add to 100% in cases where categories were not mutually exclusive

## Results

An electronic search yielded a total of 695 titles and abstracts which were judged to be potentially relevant based on title and abstract reading. Of these, 160 records were excluded for being duplicates and 2 were published before 1990. Full texts and abstracts of the remaining 533 articles were reviewed where 480 were excluded because they were not related to mental health. A total of the remaining 53 full-text articles were assessed for eligibility where 23 articles were excluded because they were either non-CA and non-DCEs or non-peer reviewed. A total 30 articles 30 were ultimately reviewed based on their satisfaction of inclusion criteria.

**Fig. 1:**
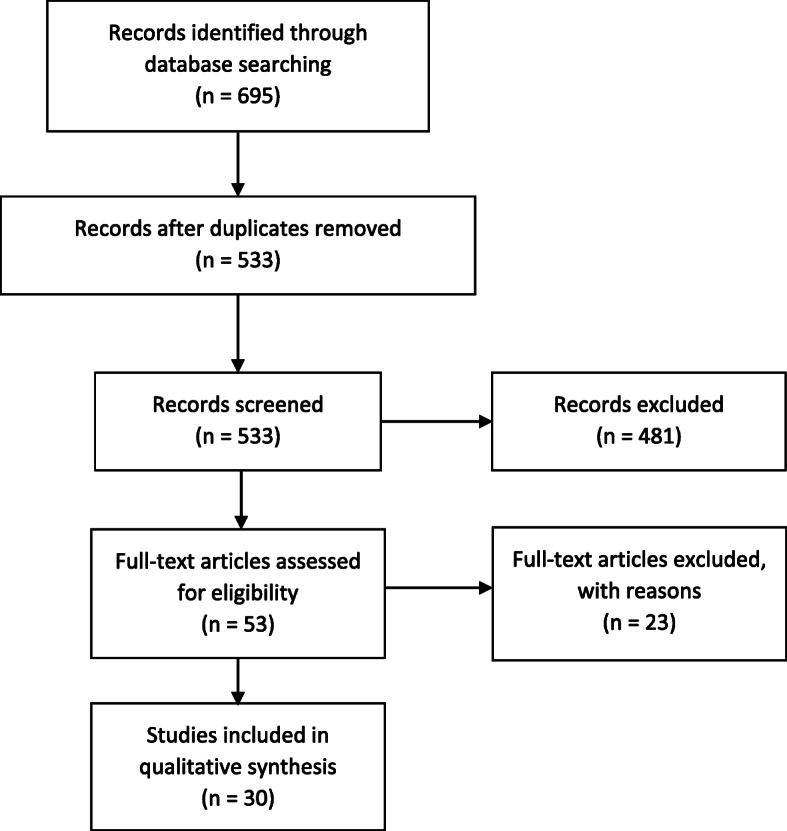
Scoping review flow diagram

A flow chart through the different steps of study selection is provided in Fig. 1.

### Conjoint analysis/discrete choice experiment characteristics

#### Study location and year

The studies included were published between 2000 and 2018, the majority (21/30, 70%) of which were published since 2010. Most studies were conducted in the United States (11/30, 37%) or Canada (10/30, 33%), and all were conducted in high-income settings (Germany: 4/30, 13%, UK: 3/30, 10%, Japan: 2/30, 7%) (Table 3).

#### Study populations

Studies most frequently elicited preferences from patient and prospective patient populations (21/30, 70%), others sought preferences from parents of children requiring mental health services (7/30, 23%), and few sought mental health providers and administrators (4/30, 13%). Some studies included multiple population types. Source populations for the studies ranged widely, with some studies recruiting participants directly from waiting rooms and outpatient health facilities [28–36], some from inpatient services [37–40], some querying university students [41–44], some recruiting from service waitlists (such as those waiting initiation of a service in the Canadian national health system) [45–51], others from provider databases [52] or internet-based health community [15, 53, 54].

#### Mental health issues, services, and attributes investigated

Nearly half of the studies sought preferences for mental health services generally without focus on a particular issue or disorder (14/30, 47%). A quarter focused on preferences for unipolar depression services (8/30, 27%), and fewer focused on other mental health issues (attention deficit hyperactivity disorder: [3/30, 10%], addiction/substance use disorder [2/30, 7%], dementia [2/30, 7%], and bipolar disorder [1/30, 3%]). The mental health services of focus for the included studies ranged widely with over half (17/30, 57%) seeking stakeholder preferences for attributes of mental health care and treatment, and others focused on choices for health messages and information (2/30, 7%), prevention and early intervention services (2/30, 7%), child mental health interventions (4/30, 13%) campus-, school-, and community-based programs (2/30, 7%). One study sought preferences for psychosocial support services (1/30, 3%), one for genetic testing services for dementia (1/30, 7%), and another one for pharmacologic attribute preferences (1/30, 7%). Individual attributes assessed were extremely variable and ranged widely across studies to make further generalizations but depression remains a commonly studied condition.

Due to variability in stakeholder populations assessed, mental health issues explored, and attributes investigated in these CA and DCE studies, we did not synthesize information about patient and provider preferences identified within the CA and DCE studies. Through our systematic review, we aim to facilitate greater understanding of the design and application of CA and DCE studies for use in mental health care settings, thus we focused our results on practical aspects of existing studies. Across the 30 studies included from the last 20 years, we saw encouraging evidence of more recent CA and DCEs building upon methodologic and analytic experience from prior CA and DCEs applied to mental health topics, across varied populations. By identifying this rapidly expanded collection of CA and DCEs applied to mental health, we aim to amplify this trend such that future studies are able to build off of the knowledge accumulated over the past 20 years, expanding the application of CA and DCEs to new populations and settings.

#### Methodologic design applied to conjoint analysis and discrete choice experiments

CA and DCEs were employed with nearly equal proportion across the studies included (CA: 16/30, 53%, DCEs: 14/30, 47%) (see Table 4). Prior to developing the CA or DCE, 70% (21/30) of studies conducted qualitative exploration among patients, 50% (15/30) conducted quantitative exploration, and 43% (13/20) performed literature, or policy qualitative exploration among policy makers (3%) (Table 4). About half of studies (53%) employed ternary choice types, while others used binary (40%), or did not specify (13%). The number of attributes explored ranged from three to more than eight, yet the most often used number was more than 8 (40%) or 4 (37%). Studies most frequently posed more than 15 choices to each participant (33%), while the second most frequent number of choices was 5 or fewer (27%). Self-completed questionnaires were the most common form of administering CA and DCEs (80%), while five studies administered questionnaires by a study staff member. Sample sizes for the studies ranged from 29 to 2469, with 27% (8/30) of studies having 100 participants or fewer, 37% (11/30) having sample sizes between 101 and 300 and 33% (10/30) having over 300 participants.

The majority of CA and DCEs (57%) employed main effects and interactions in their study design plans. The most common methodologic approach to designing the choice tasks was use of orthogonal design with Bayesian analysis. Across the 30 studies, the total number of choice tasks posed within CA and DCEs ranged from 10 to over 150. Half of the analyses (15/30, 50%) utilized Sawtooth software, while SPSS was the second most-utilized statistical software (20%). Other analyses utilized SAS (13%), Stata (3%), R (3%), and many studies used multiple of the aforementioned statistical packages.

Similarly, most studies utilized multiple statistical analysis methods with the most frequently used method as logistic regression (12/30, 40%), latent class analysis as the second most used (10/30, 33%), hierarchical Bayes estimation methods were also commonly used (8/30, 27%). Other methods included ordinary least squares regression (6/30, 20%), chi-squared, ANOVA, and MANOVA tests (7/30, 23%), and ordered probit regression (4/30, 13%).

## Discussion

Our scoping review of CA and DCEs attempted to elicit stakeholder preferences and individual level service needs and demand for mental health services. We summarize the use of these preference elicitation methods to date towards finding solutions towards mental health service design and management given the increasing global health burden of mental health disorders [55]. We identified few (*n* = 30) applications of these methods in this context and highlighted depression services as the mental health disorder toward which they have been most frequently utilized. All existing studies took place in high-income settings, showcasing a gap in current application and an opportunity for expansion to low- and middle-income settings. Such settings may face a scarcity of mental health resources such that prioritization based on patient-centered and provider-informed preferences could aid in tailoring services to optimize access and acceptability. Further, applications to date have mostly focused on adult mental health care and treatment, with fewer studies focused on child health. Two studies focused on preferences from university students highlighting potential utility in seeking mental health preferences among adolescent and young adult groups – an age category at higher risk for mental health issues globally and a demographic for whom mental health promotion and prevention services are important. Our results add to the limited literature regarding an appraisal of well-developed methods to improve patient-centeredness of mental health services using rigorous sequential mixed methods. Existing evidence demonstrates feasibility and increasing interest in seeking stakeholder preferences for mental health services, and can be used to inform future studies which expand the application of these methods to other contexts and populations facing mental health problems.

### Potential of CA and DCEs in mental health research

The need to address behavioral and psychosocial problems globally is more urgent than ever and is gaining recognition within global health goal-setting such as health systems strengthening to address the non-communicable disease burden (including mental disorders) within the Sustainable Development Goals [5]. Patient and provider preference elicitation to inform intervention development and evaluation should be considered an integral component of quality of care and service development globally. Recognizing our patients and community stakeholders as experts in their own treatment and service needs empowers them to take part in designing care that is acceptable, appropriate, and desirable. Service areas such as psychological and psychiatric services which may be underdeveloped and stigmatized in many settings could especially benefit from patient-informed alternatives, which may encourage utilization of services and, ultimately, alleviation of mental health burden. Such methods might also help us develop programs and services that may mitigate stigma and routinely experienced barriers to care. Here is an example of a DCE study that could give pointers to what patients might look forward to and inconveniences might be willing to overlook A study from South Africa echoed a similar sentiment based on a DCE looking at public health care in which they found that communities were prepared to tolerate public sector health service characteristics such as a long waiting time, poor staff attitudes and lack of direct access to doctors if they received the medicine they need, a thorough examination and a clear explanation of the diagnosis and prescribed treatment from health professionals [56].

#### Adapting and tailoring mental health interventions based on patient preferences

Conjoint methods sharpen the focus on “what it is about treatment” that drives preferences and provides specific guideposts for how to design packages of treatment that are patient-centered. A number of studies covered depression and psychosocial support [15, 28–33, 35, 37–40, 42, 43, 53, 54, 57–59] from the premise that theoretical assimilation of intervention or treatment preference characteristics might vary from real life choices and concerns. A DCE is a quantitative tool that measures the weight of different factors that affect a decision. Participants are presented with two hypothetical scenarios to choose between. Some studies found that the patients expected more personal support from healthcare providers, including flexible working hours and higher quality of patient-provider relationships [60]. Preference elicitation is a key component of the treatment engagement process, improving understanding of which treatment types or strategies best support the priorities of the patient population and, thus improve their outcomes while bolstering their connection to care. Choices prioritized by patients for mental health services may illuminate their own conceptualizations of mental health issues which may highlight opportunities to utilize key health messages for psychotherapeutic interventions. Studies identified in this scoping review showcase that low literacy populations can be effectively included in preference elicitation exercises using simple visualizations and choice tasks that are broken down into basic categories. Other studies demonstrated that patient-preferences identified with conjoint analysis or discrete choice experiments could be used in conjunction with information about existing services, input from healthcare professionals, and qualitative interviews with patients to arrive at a more comprehensive plan for intervention and service development. Importantly, these methods may help serve the needs of diverse populations by informing appropriate and effective mental health services tailored to unique sub-groups. Discrete choice experiments and conjoint analysis might be useful to inform the development of tools to assist shared decision making in psychiatry [61]. Similar ideas were expressed in a DCE carried in Tanzania focused on maternal health care which found that care quality, both technical and interpersonal, was more important than clinic inputs such as equipment and cleanliness [62].

#### Incorporating preferences of mental health specialists for sustainable capacity and leadership

Our findings identified examples where conjoint analysis and discrete choice experiments were used to identify nuanced barriers and needs for capacity building among health providers and mental health specialists [33, 36, 52, 63]. Implementation of evidence-based psychological and psychiatric interventions is complex, thus using quantitative preference elicitation methods to understand service provision processes at the administration, health system, and provider levels could streamline the complexity. Studies from our scoping review identified the desire from mental health providers and administrators for enhanced supervisory support, local decision control in treatment approaches, improved training in psychopathology, more leadership and flexibility in implementation processes, and further training opportunities. Overall, these methods may offer opportunities to improve service evaluation and health system feedback loops via input from health providers and administrators to improve quality of mental health service provision.

#### Strengthening health systems to deliver patient-centered mental health services

As the need for effective mental health services is increasingly recognized globally, methods to ensure that such services are relevant and responsive to the needs of patient populations are essential. Rigorous, quantitative approaches to ascertaining input from stakeholders, such as conjoint analysis and discrete choice experiments, have been specifically recommended for integrating mental health services within health systems in low- and middle-income countries [64]. Individual level patient and provider preferences that are identified and incorporated into design and implementation of mental health services synergistically strengthen provision. By seeking contributions from populations served, use of these methods improves appropriateness and desirability of services which may improve equity in mental health care. Additionally, the development of knowledge transfer strategies that align the preferences of professionals with those of the families they serve will go a long way in strengthening the system and services [65].

### Limitations

This scoping review was limited to peer-reviewed, published literature; thus, we did not account for conjoint analyses or discrete choice experiments for mental health service preferences reported in other sources. Further, we limited our review to studies available in English language, thus we may have missed findings from other settings published in other languages. Despite these limitations, we feel we were able to achieve our goal of scoping applications of conjoint analysis and DCEs for preference elicitation regarding mental health services through this review.

## Conclusions and future directions

The objective of this scoping review was to describe existing applications of conjoint analysis and discrete choice experiments for eliciting stakeholder preferences, individual patient and provider level for mental health services within published literature. We found that conjoint analysis and discrete choice experiments have been increasingly used over the past 20 years to identify preferences from diverse populations and a range of mental health issues and services. All conjoint analyses identified for this scoping review were performed within high-income countries, yet a few were performed within low-income populations in those settings. Conjoint analysis and discrete choice experiments have been shown as effective methods for eliciting preferences for mental health services within diverse settings, illustrating a promising approach to increasing patient-centered mental health care. Future applications of such methods should be performed within low- and middle-income countries to assess the performance of this methodology within settings where patient involvement in care is traditionally low and appropriate mental health services are lacking. Ultimately, we assert that application of preference elicitation methods such as conjoint analysis and discrete choice experiments should be applied to mental health services among populations globally to expand utilization and reduce mental health burden.

## Supplementary Information

**Additional file 1: Supplementary Table 1.** Search terms per database searched.

## Data Availability

The datasets used and/or analyzed during the current study available from the corresponding author on reasonable request.

## Abbreviations

CA: Conjoint analysis
DCE: Discrete choice experiments
HIV: Human immunodeficiency virus
SDGs: Sustainable Development Goals
W.H.O: World Health Organization
AA-HA: Accelerated Action for Health of Adolescents
PRISMA-ScR: Preferred Reporting Items for Systematic reviews and Meta-Analyses extension for Scoping Reviews
CINAHL: Cumulative Index to Nursing and Allied Health Literature
EMBASE: Excerpta Medica dataBASE
ANOVA: Analysis of Variance
MANOVA: Multivariate analysis of variance
