# Supplementary material for: Mental health service preferences of patients and providers: a scoping review of conjoint analysis and discrete choice experiments from global public health literature over the last 20 years (1999–2019)
Source: BMC Health Serv Res. 2021 Jun 18;21:589. doi: 10.1186/s12913-021-06499-w (PMC8214295; doi:10.1186/s12913-021-06499-w)
Supplement: Supplementary file 1 — Additional file 1: Supplementary Table 1. Search terms per database searched. [file 12913_2021_6499_MOESM1_ESM.docx]

**Supplementary Table 1. Search terms per database searched**

| **PubMed** | **CINAHL** | **PsychInfo** | **EMBASE** | **Cochrane** | **Web of Science** |
| --- | --- | --- | --- | --- | --- |
| conjoint[tiab] | conjoint | (TI conjoint OR AB conjoint) | (conjoint:ti,ab) | conjoint | conjoint |
| AND | AND | AND | AND | AND | AND |
| (analysis[tiab] OR analyses[tiab] OR design*[tw] OR experiment*[tw]) | (analysis OR analyses OR design* OR experiment*) | (TI analysis OR AB analysis OR TI analyses OR AB analyses OR TI design* OR AB design* OR TI experiment* OR AB experiment*) | (analysis:ti,ab OR analyses:ti,ab OR design* OR experiment*) | (analysis OR analyses OR design* OR experiment*) | (analysis OR analyses OR design* OR experiment*) |
| AND | AND | AND | AND | AND | AND |
| (mental health[tw] OR depression[tw]  OR depressive[tw] OR anxiety[tw] OR mental disorders[mh] OR mental health services[mh]) | (choice* OR choose OR choosing OR prefer OR preference* OR patient centered) | (choice* OR TI choose OR AB choose OR TI choosing OR AB choosing OR TI prefer OR AB prefer OR TI preference* OR AB preference* OR "patient centered") | (choice*:ti,ab,de OR choose:ti,ab OR choosing:ti,ab OR prefer:ti,ab OR preference*:ti,ab,de OR 'patient centered':ti,ab,de) | (choice* OR choose OR choosing OR prefer OR preference* OR patient centered) | (choice* OR choose OR choosing OR prefer OR preference* OR patient centered) |
| AND | AND | AND | AND | AND | AND |
| (choice*[tw] OR choose[tiab] OR choosing[tiab] OR prefer[tiab] OR preference*[tw] OR "patient centered"[tw]) | (MH health personnel+ OR provider* OR practitioner* OR physician* OR health care worker* OR healthcare worker* OR health educator* OR administrator* OR clinician* OR nurses OR dentists OR personnel* OR pharmacists OR workforce OR respondent OR respondents OR respondents OR participant* OR patient OR patients OR outpatients OR men OR male OR women OR female OR parents OR caregiver*) | (DE "Health Personnel" OR DE "Allied Health Personnel" OR DE "Caregivers" OR DE "Medical Personnel" OR DE "Mental Health Personnel" OR provider* OR practitioner* OR physician* OR "health care worker*" OR "healthcare worker*" OR "health educator*" OR administrator* OR clinician* OR nurses OR dentists OR personnel* OR TI "pharmacists OR workforce" OR AB "pharmacists OR workforce" OR TI respondent OR AB respondent OR TI respondents OR AB respondents OR participant* OR patient OR patients OR outpatients OR TI men OR AB men OR TI male OR AB male OR TI women OR AB women OR TI female OR AB female OR TI parents OR AB parents OR caregiver*) | ('health care personnel'/exp OR provider*:ti,ab,de OR practitioner*:ti,ab,de OR physician*:ti,ab,de OR "health care worker*":ti,ab,de OR "healthcare worker*":ti,ab,de OR "health educator*":ti,ab,de OR administrator*:ti,ab,de OR clinician*:ti,ab,de OR nurses:ti,ab,de OR dentists:ti,ab,de OR personnel*:ti,ab,de OR pharmacists:ti,ab,de OR workforce:ti,ab OR respondent:ti,ab OR respondents:ti,ab OR participant*:ti,ab OR patient:ti,ab,de OR patients:ti,ab,de OR outpatients:ti,ab,de OR men:ti,ab OR male:ti,ab OR women:ti,ab OR female:ti,ab OR parents:ti,ab OR caregiver*:ti,ab,de) | (health personnel OR provider* OR practitioner* OR physician* OR health care worker* OR healthcare worker* OR health educator* OR administrator* OR clinician* OR nurses OR dentists OR personnel* OR pharmacists OR workforce OR respondent OR respondents OR participant* OR patient OR patients OR outpatients OR men OR male OR women OR female OR parents OR caregiver*) | (health personnel OR provider* OR practitioner* OR physician* OR health care worker* OR healthcare worker* OR health educator* OR administrator* OR clinician* OR nurses OR dentists OR personnel* OR pharmacists OR workforce OR respondent OR respondents OR participant* OR patient OR patients OR outpatients OR men OR male OR women OR female OR parents OR caregiver*) |
| AND |  | AND | AND |  |  |
| (health personnel[mh] OR provider*[tw] OR practitioner*[tw] OR physician*[tw] OR health care worker*[tw] OR healthcare worker*[tw] OR health educator*[tw] OR administrator*[tw] OR clinician*[tw] OR counselor* OR psychiatrist* OR nurses[tw] OR dentists[tw] OR personnel*[tw] OR pharmacists[tiab] ORd workforce[tiab] OR respondent[tiab] OR respondents[tiab] OR participant*[tw] OR patient[tw] OR patients[tw] OR outpatients[tw] OR men[tiab] OR male[tiab] OR women[tiab] OR female[tiab] OR girl*[tw] OR boy*[tw] OR participant*[tw] OR parents[tiab] OR caregiver*[tw]) |  | PY 1990-2019 | [1990-2019]/py |  |  |
|  |  |  | AND |  |  |
|  |  |  | [embase]/lim NOT ([embase]/lim AND [medline]/lim) |  |  |
|  |  |  | AND |  |  |
|  |  |  | ([note]/lim OR [conference abstract]/lim OR [editorial]/lim) |  |  |
